# Supplementary material for: Characterization of Adherent-Invasive Escherichia coli (AIEC) Outer Membrane Proteins Provides Potential Molecular Markers to Screen Putative AIEC Strains
Source: Int J Mol Sci. 2022 Aug 12;23(16):9005. doi: 10.3390/ijms23169005 (PMC9409007; doi:10.3390/ijms23169005)
Supplement: Supplementary file 1 [file ijms-23-09005-s001.zip › Supplementary Tables S3-S7.pdf]

Supplementary material

Characterization of Adherent-invasive *Escherichia coli* (AIEC) outer membrane proteins provides potential molecular markers to screen putative AIEC strains

Table S1. Characteristics, origin, and antimicrobial profile of the 16 *E. coli* strains isolated from patients with Crohn's disease included in this study

Information available in an attached MS Excel file

Table S2. Accession numbers of *E. coli* genomic sequences used in this study

Information available in an attached MS Excel file

Table S3. Frequency of the presence of the 16 OMPs identified by MALDI-TOF/TOF in the 7 AIEC strains characterized in this study.

AIEC strains	OMPs															
	**ChuA**	**CirA**	**FitA**	**FepA**	**LgC**	**IrpC**	**OmpT**	**NmpC**	**OmpA**	**EefC/**	**TolC**	**BtuB**	**Dps**	**LptD**	**Tsx**	**EF-TU**
**4C01**	+	+	+	+	-	+	+	-	+	+	+	+	+	-	+	+
**4I01**	+	+	+	+	-	+	+	-	-	+	+	+	-	-	+	+
**5C01**	+	+	+	+	+	+	+	+	-	-	+	+	+	+	+	+
**6I09**	+	+	+	+	+	+	+	+	+	+	+	+	+	+	+	+
**9C01**	+	+	+	+	+	+	+	+	+	+	+	+	+	+	+	+
**10C01**	+	+	+	+	+	+	+	+	+	+	+	+	+	+	+	+
**18I08**	+	+	+	+	+	+	+	+	+	+	+	+	+	+	+	+
Frequency (%)	100	100	100	100	71	100	100	71	71	86	100	100	86	71	100	100

Table S4. Presence of the 16 OMPs of interest in AIEC and commensal *E. coli* strains based on the analysis of sequences of their complete genomes in GenBank by BLASTp*

Protein	GenBank Accession	AIEC strains								Commensal *E. coli* strains			
		**LF82**	**HM605**	**NRG857**	**UM146**	**541-1**	**541-15**	**576-1**	**%**	**HS**	**IAI1a**	**SE11**	**%**
Hemo/iron group receptor, ChuA	CAP77956.1	+	+	+	+	-	-	+	71.4	-	-	-	0
Siderophore, colicin, microcin receptor, CirA	CAP76657.1	+	+	+	+	+	+	-	85.7	+	+	+	100
Iron-ferrichrome receptor, FitA	CAP77503.1	+	+	+	+	-	-	-	57.1	-	-	-	0
Ferrienterobactin receptor, FepA	CAP75084.1	+	+	+	+	+	+	+	100	+	+	+	100
Nucleoside receptor, phage T6 and colicin K	CAP74945.1	+	+	+	+	+	+	+	100	+	+	+	100
Outer membrane protein, NmpC	CAP75656.1	+	-	+	+	-	-	-	42.8	-	-	+	33
LPS-assembly protein, LptD	CAP74626.1	+	+	+	+	+	+	+	100	+	+	+	100
Outer membrane protein, TolC	CAP77509.1	+	+	+	+	+	+	+	100	+	+	+	100
Outer membrane channel, EefC/NodT	CAP75839.1	+	+	+	+	-	-	-	57.1	-	-	-	0
Vitamin B12 transporter, BtuB	CAP78431.1	+	+	+	+	+	+	+	100	+	+	+	100
Yersiniabactin/pesticin receptor, IrpC	CAP76464.1	+	+	+	+	-	+	+	85.7	-	-	-	0
Protease OmpT	CAP75065.1	+	+	+	+	-	-	-	57.1	-	-	+	33
Elongation factor, Tu EF-Tu	CAP77791.1	+	+	+	+	+	+	+	100	+	+	+	100
Potential iron receptor, Lgc	ADN70802.1	-	-	-	+	+	+	-	42.8	-	-	-	0
DNA-protecting protein, Dps	CAP75282.1	+	+	+	+	+	+	+	100	+	+	+	100
Outer membrane protein, OmpA	CAP75420.1	+	+	+	+	+	+	+	100	+	+	+	100

* The proteins that were more frequent in AIEC strains than in commensal *E. coli* strains are marked in gray.

Table S5. Detection by PCR of the genes that code for the OMPs of interest in 8 commensal *E. coli* strains.

	fitA	*irpC*	*ompT*	*chuA*	*eefC/nodT*	*nmpC*	*lgC*
**strain 1**	-	-	-	-	-	-	-
**strain 2**	-	-	-	-	-	-	-
**strain 3**	+	+	+	+	+	+	-
**strain 4**	-	-	-	-	-	-	-
**strain 5**	-	+	-	-	-	+	-
**strain 6**	-	+	-	-	-	+	-
**strain 7**	-	+	-	-	-	+	-
**strain 8**	-	+	-	-	-	+	-
**Frequency**	1/8 (13%)	5/8 (55%)	1/8 (13%)	1/8 (13%)	1/8 (13%)	5/8 (55%)	0/8 (0%)

Table S6. Binary logistic regression model evaluating the *chuA* + *eefC* + *fitA* genes as molecular markers for AIEC identification*

Equation variables				
	**Estimate**	***p*-value**	**Odd ratio**	**95% CI**
chuA	-3.258	0.0013	0.03846	0.004190 -0.2542
eefC* + *fitA	0.5878	0.5488	1.800	0.2782 – 15.33
**Intercept**	2.565	0.0001	13.00	5.321 – 42.97
**Classification performance**				
	**Predicted**			
**Observed**	AIEC	Non-AIEC	Total	% Correctly classified
**AIEC**	14	4	18	77.8 (Sensitivity)
**Non-AIEC**	11	52	63	82.5 (Specificity)
**Total**	25	56	81	81.5 (Accuracy)

* This analysis was made using GraphPad Prism vs. 9.1.0.

Table S7. Cross-validation of gene markers (*chuA, eefC* and *fitA*) for AIEC identification by Random Forest and Naive Bayes methods *

Method	Average AUC	Sensitivity (%)	Specificity (%)	CA (%)
**RF**	0.82	0.81	0.49	0.81
**NB**	0.81	0.84	0.72	0.84

* Random Forest (RF) and Naive Bayes (NB) classification algorithms were implemented using the Orange data mining suite, V.3.27.0 (http://orange.biolab.si). RF is an ensemble method using decision trees and NB is a generative model. 66% of the 81 strains that were used in the phylogenetic analysis were the training set and the rest were used as the test set with the five-fold cross-validation method. AUC, Area under the curve. CA, Classification accuracy measures the ratio of the correct predictions to the total number of instances evaluated.
